# Heart Rate Variability Duration: Expanding the Ability of Wearable Technology to Improve Outpatient Monitoring?

**DOI:** 10.3389/fpsyt.2021.682553

**Published:** 2021-06-15

**Authors:** David C. Sheridan, Karyssa N. Domingo, Ryan Dehart, Steven D. Baker

**Affiliations:** ^1^Department of Emergency Medicine, Oregon Health & Science University, Portland, OR, United States; ^2^Center of Policy and Research in Emergency Medicine, Oregon Health & Science University, Portland, OR, United States

**Keywords:** heart rate variability, suicidality, wearable technology, anxiety, outpatient

## Abstract

Heart rate variability (HRV) evaluates beat-to-beat interval (BBI) differences and is a suggested marker of the autonomic nervous system with diagnostic/monitoring capabilities in mental health; especially parasympathetic measures. The standard duration for short-term HRV analysis ranges from 24 h down to 5-min. However, wearable technology, mainly wrist devices, have large amounts of motion at times resulting in need for shorter duration of monitoring. The objective of this study was to evaluate the correlation between 1 and 5 min segments of continuous HRV data collected simultaneously on the same patient. Subjects wore a patch electrocardiograph (Cardea Solo, Inc.) over a 1–7 day period. For every consecutive hour the patch was worn, we selected a 5-min, artifact-free electrocardiogram segment. HRV metric calculation was performed to the entire 5-min segment and the first 1-min from this same 5-min segment. There were 492 h of electrocardiogram data collected allowing calculation of 492 5 min and 1 min segments. 1 min segments of data showed good correlation to 5 min segments in both time and frequency domains: root mean square of successive difference (RMSSD) (*R* = 0.92), high frequency component (HF) (*R* = 0.90), low frequency component (LF) (*R* = 0.71), and standard deviation of NN intervals (SDNN) (*R* = 0.63). Mental health research focused on parasympathetic HRV metrics, HF and RMSSD, may be accomplished through smaller time windows of recording, making wearable technology possible for monitoring.

## Introduction

Heart rate variability (HRV) evaluates the beat-to-beat time interval (BBI) differences during the cardiac cycle. HRV has been shown to correlate with several medical conditions (1, 2). One area of medicine with particular interest in HRV is mental health. One recent study found there were over 1.2 million annual emergency department visits by adolescents in the United States for suicidality. This has doubled from the most recent decade (3). Even worse, up to 20% of adolescents will have a repeat suicide attempt within 12 months from an initial attempt and almost half of patients will have a second emergency department visit after their first for mental health (4, 5). The exponential increase in mental health crises over the past decade is overwhelming emergency departments. This has created a critical need for innovative approaches to early identification and intervention. HRV has been postulated in prior mental health research to reflect the balance of the autonomic nervous system. This has been proposed to be dysregulated in conditions such as suicidality; in particular a downregulation of the parasympathetic nervous system (6). Although HRV has been around for a long time, it has gained favor recently with the expansion of technology and mobile health applications specific to mental health (7). The ability to accurately measure HRV therefore has broad implication for mental healthcare.

HRV has traditionally been obtained from chest wall electrocardiography (ECG) and norms have been described previously (8). However, advancements in wearable technology like smartwatches can detect HRV through photoplethysmography (PPG). This obtains a plethysmogram optically by measuring microcirculatory changes in blood volume for BBI detection (9, 10). PPG introduces significant potential in a socially accepted platform: wrist-worn technology. This platform may have the potential for patients with mental health to monitor themselves at home. As the technology detects changes in the autonomic nervous system, patients can be notified to practice their coping mechanisms and therapies developed with their provider. HRV analysis utilizes small time changes to calculate specific time and frequency metrics over extended periods. One unique challenge to wrist-worn devices is motion artifact and its impact on being able to obtain long durations of accurate continuous BBI data. Studies show that HRV metrics can tolerate small shifts or loss of data caused by motion artifacts (11). Traditionally, the minimum recording for HRV metrics has been 5 min (12). This timeframe may be challenging for wrist technology to accurately measure HRV metrics aimed at mental health including parasympathetic measures of the high frequency component (HF) and root mean square difference (RMSSD) due to motion.

This study aims to determine if 1-min segments of gold standard ECG-derived HRV data correlate to 5-min segments. This data may establish a new standard for measurement in future HRV analysis studies, particularly to define procedures used on wearable technologies such as wrist-worn devices that are susceptible to occasional motion artifact.

## Materials and Methods

This was a prospective convenience sample of individuals of any age without a formal mental health or cardiac diagnosis wearing a patch ECG technology (Cardiac Insight, Cardea Solo). Subjects were recruited directly from the study team or healthy individuals if >13 years old, without daily medications or comorbid conditions including smoking, and not being evaluated in the emergency department for an acute complaint. This population was chosen to ensure there were not confounders related to mental health or cardiac disease affecting BBIs and allow for normal daily activities. This study underwent institutional review board approval at the study site (Oregon Health & Science University IRB study 16864). The patch ECG was placed over the left upper chest as described by the Cardiac Insight instructions. Subjects wore patches for 24–168 h with a goal of 96 h continuously during the day and night. BBIs were obtained directly from the patch ECG waveform data. Python 3 HRV-Analysis package (version 1.3) available from pypi.org then processed this data. The first steps in HRV-analysis are typically detection and removal of outliers and ectopic beats. Because this study is comparing two different lengths of HRV data where removal of data might affect the statistics, the data processing first searched for 5-min segments of artifact-free HRV data, where neither outliers nor ectopic beats were detected. Outliers were defined as those where the BBI was outside the range of 400–1,500 ms, which reflects a heart rate above 150 or below 40 BPM. Next, time domain features and frequency domain features were calculated for each 5-min, artifact-free segment. Many 5-min segments were available for each hour, but the study required only one segment per hour. To select the one segment for each hour, the study took the next segment that was acquired at least 60 min later than the most-recently selected 5-min segment.

From each 5-min segment, a 1-min segment was extracted from the first minute of this segment. HRV metrics calculated included high-frequency component (HF), root mean square of successive difference (RMSSD), low-frequency component (LF), and standard deviation of next normal interval (SDNN); the next normal interval is similar to the BBI. The LF and HF bands have frequencies from 0.04–0.15 Hz and 0.15–0.40 Hz, respectively. Prior literature has utilized these ranges for mental health research. For each category of HRV metrics, we calculated the correlation of the 1 and 5 min segments expressed as Pearson's Correlation Coefficients (R). The degree of correlation was categorized as excellent for an R of 0.9–1.0, good for an R of 0.7–0.89, moderate for 0.4–0.69, and low for <0.39 (13).

## Results

Continous patch ECG data was obtained for 492 h. This allowed for 492 5 min segments and 1 min segments for analysis. The patches were worn by 4 individuals, each at two unique time points. The average age was 34.5 years.

### High Frequency

**Figure 1:**
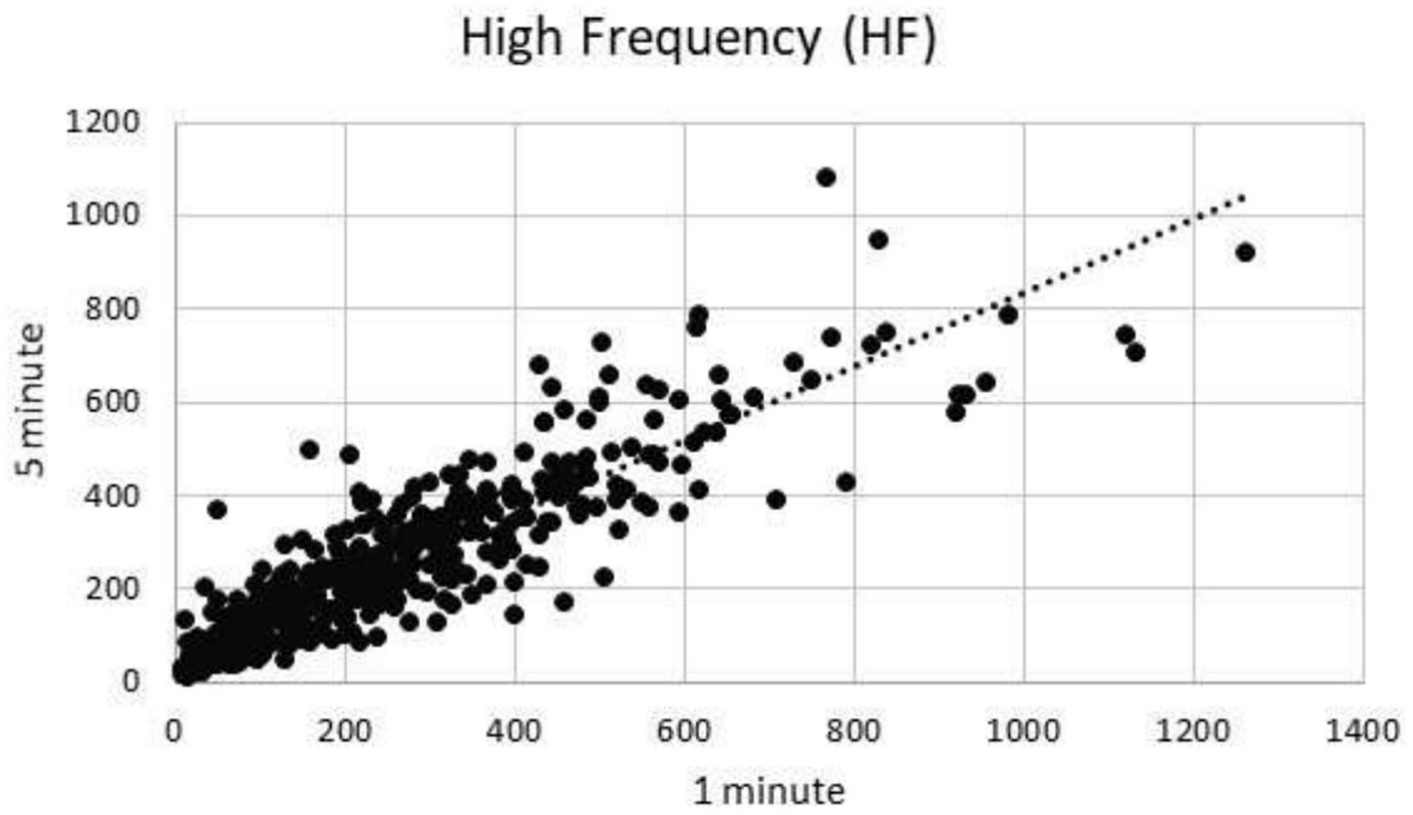
High frequency component correlation.

One minute segments of HF showed excellent correlation to 5 min segments of HF (*R* = 0.90) (Figure 1).

### Root Mean Square of Successive Differences

**Figure 2:**
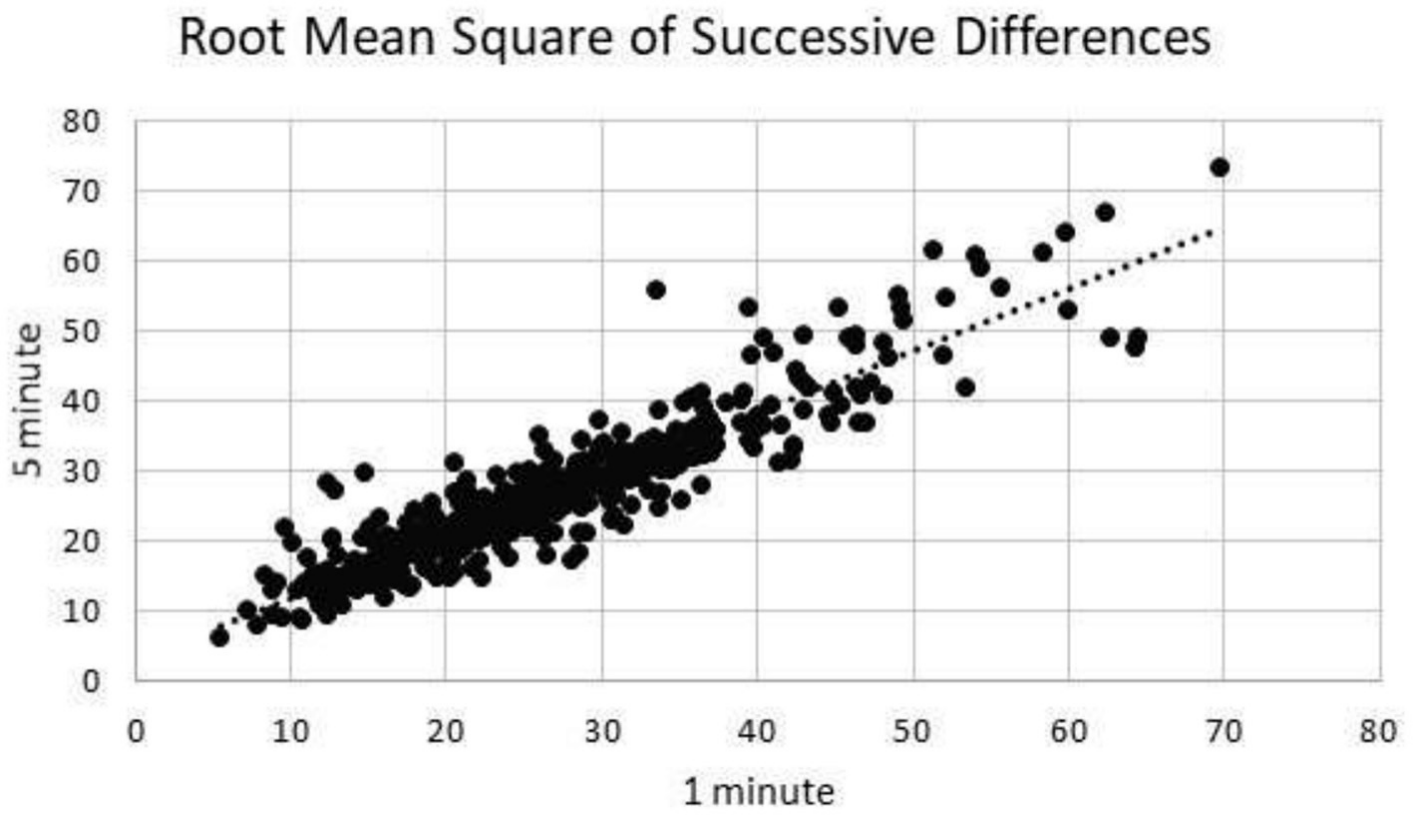
Root mean square of successive differences correlation.

One minute segments of RMSSD showed excellent correlation to 5 min segments of RMSSD (*R* = 0.92) (Figure 2).

### Low Frequency

**Figure 3:**
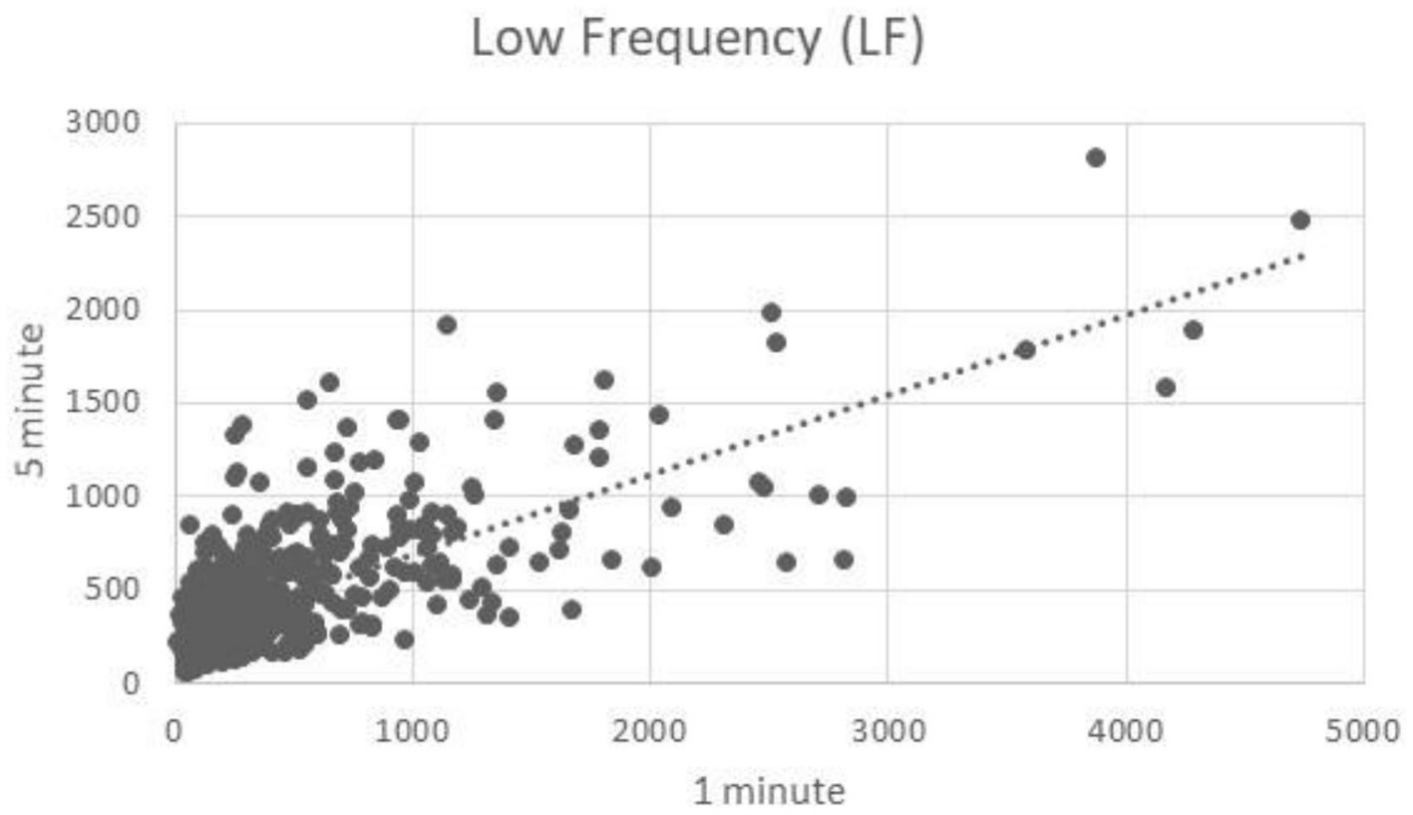
Low frequency component correlation.

One minute segments of LF showed good correlation to 5 min segments of LF (*R* = 0.71) (Figure 3).

### Standard Deviation of NN Intervals

**Figure 4:**
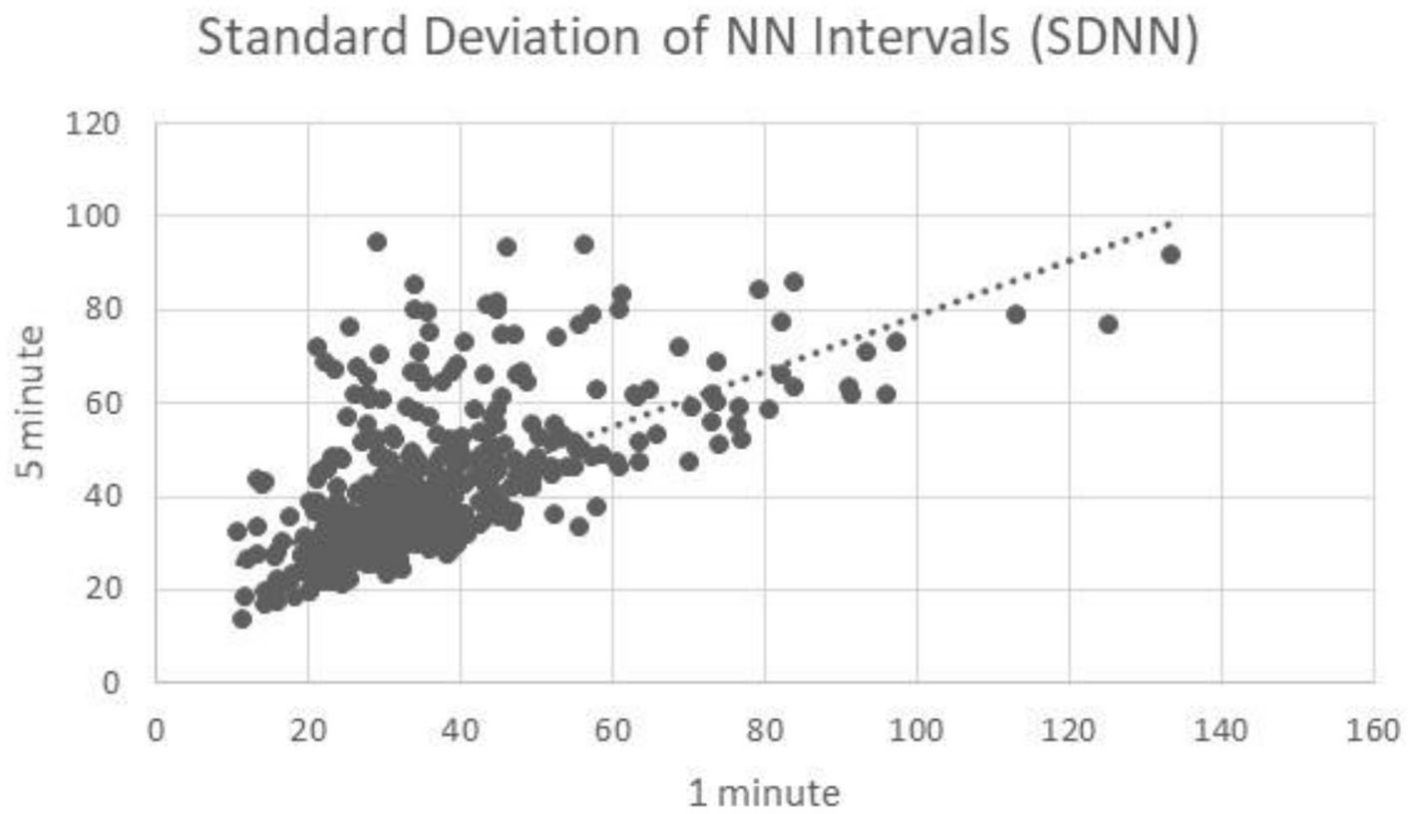
Standard deviation of NN intervals correlation.

One minute segments of SDNN showed moderate correlation to 5 min segments of HF (*R* = 0.63) (Figure 4).

## Discussion

HRV monitoring is an old metric that has provided new insight into various medical conditions including mental health (14, 15). The growing technology and platforms to collect this data now include wrist devices in the outpatient setting, but motion artifact is an issue (11). This study evaluated the correlation between 1 and 5 min segments for the most common used HRV metrics in mental health. Our results found that HF and RMSSD showed excellent correlation, LF good correlation, and SDNN moderate correlation, which is promising for future wrist-worn technology data analysis.

HRV is a metric using the BBI which is different than the heart rate measured by many applications (16). Mental health is an area of medicine that has classically not utilized significant advances in technology clinically due to its complex multifactorial pathophysiology. Yet, HRV is particularly interesting in mental health care as a reflection of the autonomic nervous system. In particular it has the ability to evaluate the homeostasis of the sympathetic and parasympathetic nervous systems; namely a dysregulation of the parasympathetic nervous system in times of stress may be a marker of escalating mental health states (17). Prior studies have utilized HRV and shown a difference in the markers of the parasympathetic nervous system between patients with a history of suicidality and those without (14, 18). Recent studies have focused on the ability of the HF and RMSSD components to evaluate patients during various situations as a marker or stress and depression (17, 19, 20). These are encouraging as they have found a decrease in the parasympathetic measures in patients with a history of suicidality or depression compared to patients without when undergoing stressful tasks or baseline measurements. This creates the possibility that HRV may function as an important measure of stress that can be monitored in the patients. Many of these studies have utilized the laboratory setting and compared groups of patients with a history of suicidality and those without.

However, mental health and suicidality have been shown to be a dynamic process that changes throughout the day and most likely requires more consistent/continuous monitoring for symptom changes (21, 22). This introduces the possibility of wearable technology to significantly improve outpatient monitoring and care delivery from the current standard assessments that administer validated questionnaires (23). PPG utilizes an optical source for changes in blood volume that reflect the cardiac cycle and thus make heart rate and BBI calculations feasible with high accuracy (9). This technology is standard now in many smartwatches on the market creating a significant opportunity for outpatient medical care. One of the largest limitation to wrist device PPG compared to standard chest wall ECG is the motion artifact that impacts long durations of continuous data. However, prior studies have shown that even having to remove small amounts of BBIs in a segment can result in no significant change in HRV metrics (11). Yet, expecting prolonged time courses of artifact-free data is unrealistic in individuals who are moving around freely. One prior study aimed to look at various durations of HRV data using machine learning and found durations <5 min to be quite accurate for detecting stress vs. non-stress in subjects (24). An addition study sought to compare the correlation between 24 h HRV segments and 5 min segments to evaluate how short of time courses are reasonable for future use (25). Similar to our results, this study evaluated the correlation between shorter and longer time frames of HRV. They found that the HF component had a strong correlation between the two sets of data (*R* = 0.817). Prior research has been published establishing norms and recommendations for monitoring durations with experimental designs in mental health studies (8, 26). These studies detail that comparing shorter to significantly longer durations or recording can confound the data. Therefore, the ability to collect short durations of HRV accurately across time settings can be very valuable as studies show it's utility. This is encouraging that separate studies had similar findings of shorter duration maintaining accuracy in the HF component, which may be the most important in future mental health studies.

The translation of this data has broad implications for medicine and mental health. The current standard of care for suicidality monitoring relies on subjective scoring tools requiring administration by a trained provider. In addition, this requires an individual in times of crisis to reach out to someone for help. Rather than this reactive approach, passive monitoring with technology can have an important role in proactive identification of crisis escalations to allow preventative treatments. Inherent confounders can be controlled for in this analysis including circadian rhythm as previously reported (27). Much research still needs to occur, but this data is encouraging for the possibility of outpatient HRV monitoring accomplished with shorter time frames to account for motion.

## Limitations

There are several limitations to this study. The first is that the study was a small cohort of healthy individuals. This eliminates confounders of underlying cardiac disease or mental health that studies show have intrinsic differences in HRV. Therefore, the goal was to collect a large number of hours of continuous 1 and 5-min simultaneous data segments to compare. A second limitation is this was a foundational study to evaluate the shorter timeframes for HRV collection, but did not evaluate a specific medical condition. This was by design as HRV was not compared between subjects, but instead just the correlation between 1 and 5 min segments. Therefore, the patient population could be heterogeneous as the study compared the correlation within each patient's measurements. This data serves to inform future studies that can evaluate HRV changes over these short time frames in different populations of patients. The objective of this study evaluated the best-studied HRV metrics for mental health, HF and RMSSD, but the most frequently obtained metrics were calculated including LF and SDNN. Because the LF band's longest period includes 25 s, only 2.4 periods are included in a 1-min data segment making this inherently more challenging to monitor with shorter time frames. However, in this study across almost 500 h of data there was still good correlation. Finally, this study included gold standard chest electrodes. It may require confirmation with a wearable wrist technology using PPG for further validation as one would expect more motion artifact in that setting.

This study found that 1-min segments of HRV (HF and RMSSD) showed excellent correlation to 5-min segments. This is encouraging for future clinical studies on outpatient mental health monitoring with wearable technology but need to be confirmed or refuted in the future.

## Data Availability Statement

The raw data supporting the conclusions of this article will be made available by the authors, without undue reservation.

## Ethics Statement

The studies involving human participants were reviewed and approved by Oregon Health and Science University. Written informed consent to participate in this study was provided by the participants' legal guardian/next of kin.

## Author Contributions

DS, SB, and KD contributed to conception and design of the study. KD and RD organized the database. KD and DS performed the statistical analysis. DS wrote the first draft of the manuscript. KD, RD, and SB wrote sections of the manuscript. All authors contributed to the article and approved the submitted version.

## Conflict of Interest

The authors declare that the research was conducted in the absence of any commercial or financial relationships that could be construed as a potential conflict of interest.
